# Dietary environmental factors shape the immune defense against *Cryptosporidium* infection

**DOI:** 10.1016/j.chom.2023.11.008

**Published:** 2023-12-04

**Authors:** Muralidhara Rao Maradana, N. Bishara Marzook, Oscar E. Diaz, Tapoka Mkandawire, Nicola Laura Diny, Ying Li, Anke Liebert, Kathleen Shah, Mauro Tolaini, Martin Kváč, Brigitta Stockinger, Adam Sateriale

**Affiliations:** 1 AhR Immunity Lab, The Francis Crick Institute, London, UK; 2 Cryptosporidiosis Lab, The Francis Crick Institute, London, UK; 3 Institute of Parasitology, TBiology Centre of the Czech Academy of Sciences, České Budějovice, Czech Republic

## Abstract

*Cryptosporidium* is a leading cause of diarrheal-related deaths in children, especially in resource-poor settings. It also targets the immunocompromised, chronically infecting people living with HIV and primary immunodeficiencies. There is no vaccine or effective treatment. Although it is known from human cases and animal models that CD4^+^ T cells play a role in curbing *Cryptosporidium*, the role of CD8^+^ T cells remains to be defined. Using a *Cryptosporidium tyzzeri* mouse model, we show that gut-resident CD8^+^ intraepithelial lymphocytes (IELs) confer resistance to parasite growth. CD8^+^ IELs express and depend on the ligand-dependent transcription factor aryl hydrocarbon receptor (AHR). AHR deficiency reduces CD8^+^ IELs, decreases their cytotoxicity, and worsens infection. Transfer of CD8^+^ IELs rescues severely immunodeficient mice from death following *Cryptosporidium* challenge. Finally, dietary supplementation of the AHR pro-ligand indole-3-carbinol in newborn mice promotes resistance to infection. Therefore, common dietary metabolites augment the host immune response to cryptosporidiosis, protecting against disease.

## Graphical abstract

**Graphical abstract:**
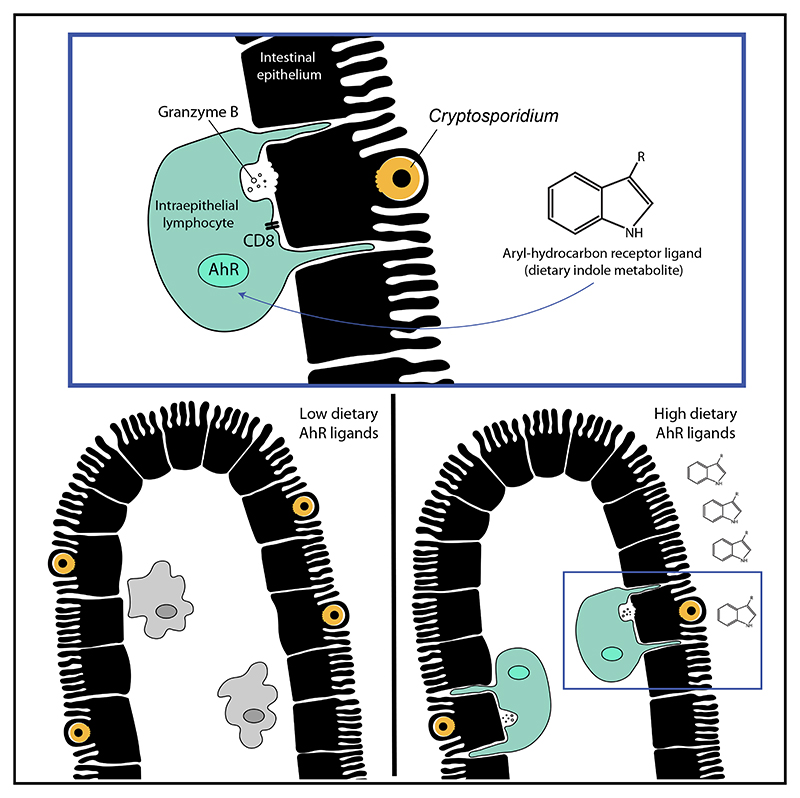

## Introduction

Diarrhea contributes to nearly 11% of early childhood mortality worldwide.^1^ *Cryptosporidium* is an apicomplexan parasite that invades epithelial cells of the small intestine,^2^ and its infections are the second-leading cause of severe diarrheal events in young children in resource-poor regions.^3^ Recurrent infections are associated with malnutrition, leading to lasting effects such as growth stunting and impaired cognitive development.^4–6^ *Cryptosporidium* is also an important opportunistic pathogen in immunocompromised individuals, such as people living with HIV, transplant, and chemotherapy recipients, and patients undergoing treatment for hemodialysis and cancer.^7,8^ Human cryptosporidiosis is usually caused by anthroponotic *Cryptosporidium hominis* or zoonotic *Cryptosporidium parvum*.^9^ There is no vaccine against cryptosporidiosis. Nitazoxanide, the only FDA approved drug to treat cryptosporidiosis, has limited efficacy in immunocompetent individuals and is ineffective in people with HIV-AIDS or malnourished individuals.^10,11^ Although some progress has been made toward the development of new therapeutics, this has been hindered by a lack of physiologically relevant model systems of cryptosporidiosis.^12,13^ We previously developed a mouse model of cryptosporidiosis with a natural mouse-infecting species, *Cryptosporidium tyzzeri*, which recapitulates the natural course of infection and intestinal pathology of human disease.^14^

Increased susceptibility of patients with primary immunodeficiencies and experimental infections of human volunteers suggest an immune system-mediated protection from cryptosporidiosis.^15^ Furthermore, most children living in endemic regions develop protective immunity to subsequent infections.^4,16^ Early studies on athymic mice revealed T cells as important regulators of *C. parvum* infection.^17^ Increased infection burden in HIV-AIDS patients with low CD4^+^ T cell counts also highlights the importance of interferon-γ (IFNγ)-producing CD4^+^ T cells.^18^ Mice lacking T cells (but not B cells) are unable to control a *C. tyzzeri* infection.^14^ Furthermore, mice without mature T and B cells, and those lacking IFNγ, cannot elicit vaccination-mediated protection during a secondary parasite infection.^14^ Mice harboring a commensal strain of *C. tyzzeri* elicit both innate and adaptive immune responses, with early evidence hinting at elevated CD8^+^ T cells in infected mice.^19^ In humans, a genetic-association study linked human leukocyte antigen (HLA) class I and II mutations with a greater incidence of infections in children aged 2–5 years, again pointing to a role for CD4^+^, as well as CD8^+^ cells in controlling *Cryptosporidium* infections.^20^ CD8^+^ T cells are highly enriched in the intestine and notably heterogeneous in phenotype and function. “Conventional” mucosal T cells express T cell receptor (TCR)αβ together with CD4 or CD8αβ as TCR coreceptors and reside in the lamina propria. “Non-conventional” mucosal T cells, expressing either TCRαβ or TCRγδ and typically also CD8αα homodimers, are prevalent in the mucosal epithelium.^21,22^ Thymic-derived natural intraepithelial lymphocytes (IELs) express CD8αα (TCRαβ^+^ CD8αα, TCRαβ^+^CD4^+^CD8αα, and TCRγδ^+^CD8αα), whereas peripherally induced IELs express CD8αβ (TCRαβ^+^ CD8αβ).^23,24^

CD8^+^ IEL cells constantly scan epithelial cells for injury or infection and are considered primary responders to epithelial damage.^25^ CD8^+^ IELs are elevated in calves and mice infected with *C. parvum*.^26,27^ Nevertheless, factors that influence CD8^+^ IEL-mediated immunity and mechanisms by which CD8^+^ IELs confer resistance to *Cryptosporidium* are unknown. Most CD8^+^ T cells exhibit a tissue-resident memory phenotype,^28^ and their maintenance depends on stimulation of the aryl hydrocarbon receptor (AHR) by endogenous ligands, such as tryptophan-derived phytochemicals, microbial metabolites, or indole derivatives.^29^ AHR is a ligand-dependent transcription factor abundantly expressed in barrier tissues, such as the gut, skin, and lungs. AHR is expressed by various cells in the gut, including barrier epithelium, endothelium, and immune cells, and is hence a key factor in maintaining gut barrier integrity.^30^ AHR deficiency or depletion of AHR ligands increases susceptibility to bacterial infection in the colon^30^ and also contributes to colon tumorigenesis.^31^

C*ryptosporidium* infects and replicates inside small intestinal epithelial cells, causing villus blunting and crypt hyperplasia and thereby significant gut damage.^14^ In human volunteers infected with *C. hominis* or *C. parvum*, increased fecal indole levels prior to infection correlated with decreased parasite burden,^32^ indicating a protective function of indoles in cryptosporidiosis through an unknown mechanism. We wondered if these indoles were working via AHR-mediated gut protection.

To test this, we created a *C. tyzzeri* reporter strain expressing luminescent and fluorescent proteins utilizing an isolate of *C. tyzzeri* from the Czech Republic (*Ct*-CR2206, shortened here to *Ct*-CR).^33^ Here, we show that in immunocompetent wild-type (WT) mice, *Ct-*CR altered epithelial differentiation and triggered an expansion of CD8^+^ IELs, which conferred protection against infection when transferred to immunodeficient mice. Immune-cell-specific deletion of AHR or the deprivation of AHR ligands in mice greatly depleted CD8^+^ IELs. Furthermore, dietary supplementation of AHR ligands to nursing mothers and their weaned pups provided prophylactic defense against infection. This highlights the opportunity for diet-based therapeutic interventions to treat this debilitating disease in humans and ruminant animals.

## Results

### *C. tyzzeri* infects the ileum and affects epithelial cell differentiation

**Figure 1:**
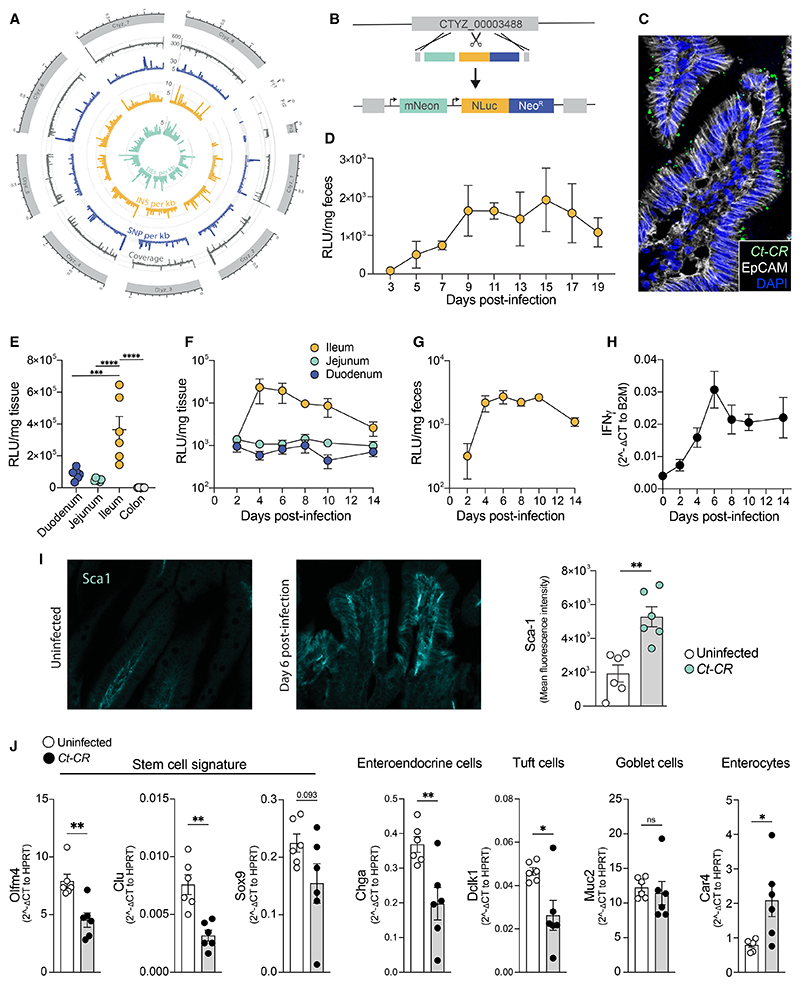
*C. tyzzeri* specifically infects the ileum and alters epithelial cell differentiation (A) Chromosome-based Circos plot mapping the newly sequenced *C. tyzzeri*-CR to the *C. tyzzeri*-UGA55 strain. Tracks from the outside-in are mean genome coverage per kb (gray), single-nucleotide polymorphism (SNP) density per kb (blue), insertions density per kb (orange), and deletions density per kb (green). (B) Schematic of the cloning strategy for the introduction of genes expressing mNeonGreen, nanoluciferase (NLuc), and neomycin phosphotransferase (Neo^R^) by Cas9-directed homology repair of the *C. tyzzeri*-CR thymidine kinase gene. (C) Immunofluorescence image showing mNeonGreen-expressing parasites in the intestinal villi from the ileal section of an immunocompromised mouse. (D) *C. tyzzeri* (*Ct*-CR) parasite burden in the feces measured by nanoluciferase assay. (E) Regional-specific *Ct-*CR parasite burden in infected wild-type (WT) mice. (F) *Ct-*CR burden in the duodenum, jejunum, and ileum. (G) Fecal levels of *Ct-*CR in infected WT mice. (H) qPCR of IFNγ in ileum tissue. (I) Sca-1-expressing epithelial cells in uninfected and *Ct-*CR-infected WT mice. (J) qPCR of purified epithelial cells for marker genes of stemness (*Olfm4, Clu*, and *Sox9*) and differentiated epithelial cells (*Chga, Dclk1, Muc2*, and *Car4*). (F–H) n = 4 per time point. (E, I, and J) Representative of at least 2 independent experiments. Each dot represents individual mice. RLU, relative luciferase units. Error bars, mean + SEM. ns-not significant, *p < 0.05, **p < 0.01, ***p < 0.001, ****p < 0.0001 as calculated by t test or one-way ANOVA with Tukey post-test.

The *C. tyzzeri* strain *Ct*-CR2206 was originally isolated from an Eastern European wild mouse (subspecies *Mus musculus musculus* [*M. m. musculus*]) in the Věrušič ky municipality of the Czech Republic.^33^ The previously characterized strain *C. tyzzeri*-UGA55 was obtained from another wild mouse sub-species (*Mus musculus domesticus*) in the United States. *Ct*-UGA55 showed considerable divergence from Eastern European wild-mouse-derived strains at the *gp60* locus, which encodes a *Cryptosporidium* surface protein commonly used for strain subtyping.^14^ We therefore sequenced the full genome of *Ct-*CR2206 (shortened here to “*Ct-*CR”) using Illumina short read sequencing. Reads were mapped using the full *Ct*-UGA55 genome as a reference (Figure 1A). In total, 13,139 single-nucleotide polymorphisms (SNPs) and 2,983 insertion-deletion (indel) events were detected between the two strains (see Table S1 for a further breakdown). To fully characterize this *M. m. musculus* derived *C. tyzzeri* strain, we genetically engineered *Ct*-CR to express the fluorescent mNeonGreen protein, along with a nanoluciferase (NLuc) and neomycin resistance cassette for easy parasite detection and transgenic selection, respectively (Figures 1B and 1C). Parasite replication over time could be tracked by assaying NLuc activity in mouse fecal pellets (Figure 1D).

*Ct*-CR infects the small intestine but not the colon (Figure 1E). To determine which specific region in the small intestine harbored parasites, we infected C57BL/6 WT mice and measured luciferase levels from duodenal, jejunal, and ileal tissue every other day up to day 14 post infection (DPI-14). This revealed the ileum to be the major site of *Ct-*CR expansion within the small intestine (Figure 1F). Despite the significant genetic drift (as evidenced by a high number of called SNPs and indels) between *Ct-CR* and the previously described *Ct*-UGA55 strain, these strains display a similar infection location and duration in mice that is parallel to what is seen in human cryptosporidiosis.^14,19^ Luciferase levels in fecal samples from the same mice reflected the parasite burden in the ileum (Figure 1G). Therefore, we used readings from fecal samples as representative measures of the *Ct-*CR burden in the mouse ileum.

IFNγ is key to controlling *Cryptosporidium* growth, for both *C. parvum*^34–36^ and the previously isolated US *C. tyzzeri* strain, UGA55.^14^ Consistently, infection with the *Ct-*CR strain led to a gradual increase in *Ifng* transcripts in the ileum (Figure 1H), suggesting that the *Ct-*CR strain is pathogenic and induces inflammation in the infected area. Sca-1 (also known as Ly6a) is a marker of mouse intestinal epithelial injury, initially identified in the context of colitis,^37^ and staining of ileal sections showed a significant increase in Sca-1 expression in the villi of infected mice (Figure 1I). To study how *Cryptosporidium* changes the epithelial cell composition in the small intestine, we purified epithelial cells at the peak of infection (DPI-6) and compared their gene expression with that of uninfected controls. Epithelial cells from infected mice showed reduced expression of the stem cell signature (*Olfm4, Clu*, and *Sox9*) and reduced expression of markers for enteroendocrine cells (*Chga*) and Tuft cells (*Dclk1*) (Figure 1J). However, no change was seen in expression of the goblet cell marker *Muc2* (Figure 1J). By contrast, there was an increase in the enterocyte marker *Car4* (Figure 1J). Therefore, the *Ct-*CR strain used in this study is pathogenic in nature and causes inflammation, epithelial cell injury, and alters the cellular composition of the small intestinal epithelium.

### Hematopoietic cell-specific aryl hydrocarbon receptor-deficient mice are susceptible to *C. tyzzeri* infection

**Figure 2:**
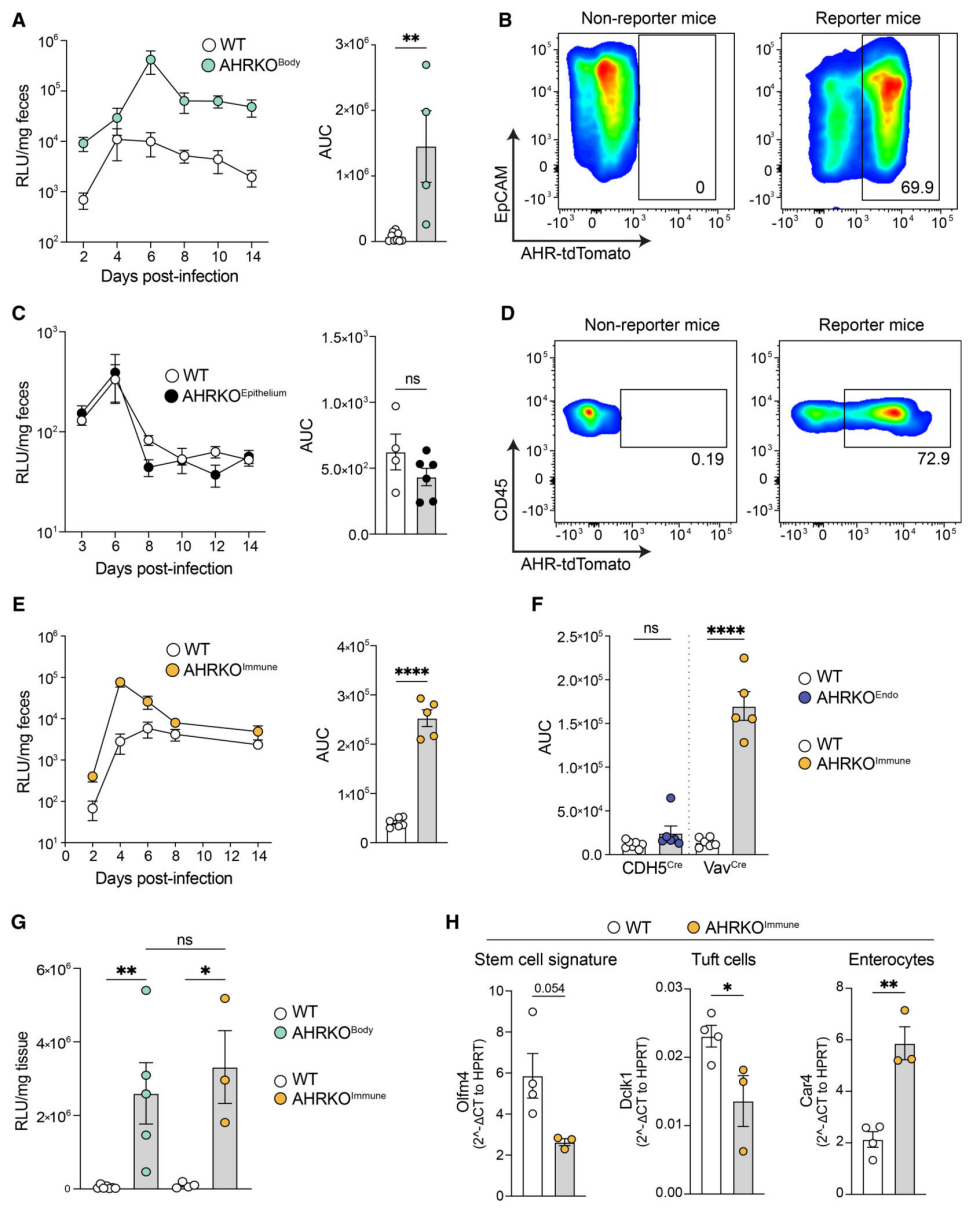
Hematopoietic cell-intrinsic AHR signaling specifically contributes to control of *C. tyzzeri* infection (A) *Ct-*CR parasite burden (left) and the area under the curve (AUC) (right) in WT and total-body AHR knockout mice (AHRKO^Body^). (B) AHR-tdTomato expression in EpCAM^+^ intestinal epithelial cells. (C) *Ct-*CR parasite growth in WT (Villin^Cre^ AHR^fl/fl^) and epithelium-specific AHR knockout mice (Villin^Cre+^ AHR^fl/fl^ termed AHRKO^Epithelium^). (D) AHR-tdTomato expression in small intestinal immune cells (CD45^+^). (E) *Ct-*CR parasite growth in WT (Vav^Cre^ AHR^fl/fl^) and immune-cell-specific AHR knockout mice (Vav^Cre+^ AHR^fl/fl^ termed AHRKO^Immune^). (F) AUC of *Ct*-CR growth in endothelial (Cdh5^Cre+^ AHR^fl/fl^ termed AHRKO^Endo^) and immune-cell-specific AHR knockout mice (AHRKO^Immune^). (G) *Ct-*CR burden at the peak of infection (DPI-6) in total-body (AHRKO^Body^) and immune-cell-specific AHR knockout mice (AHRKO^Immune^). (H) qPCR of marker genes of stem cells (*Olfm4*), Tuft cells (*Dclk1*), and enterocytes (*Car4*). (A–H) Representative results of at least 2 independent experiments. Each dot represents individual mice. RLU, relative luciferase units. Error bars, mean + SEM. ns-not significant, *p < 0.05, **p < 0.01, ****p < 0.0001 as calculated by t test or one-way ANOVA with Tukey post-test.

AHR is a transcriptional regulator of genes involved in anti-microbial defense and intestinal epithelial differentiation. It has been shown to play a protective role during colonic bacterial infection and chemically induced colon damage.^30,31,38^ To determine whether AHR executes similar disease-protective functions in the small intestine, we infected full-body AHR knockout (KO) (AHRKO^Body^) mice and co-housed WT littermate controls with *Ct-*CR. AHRKO^Body^ mice displayed increased parasite burdens from the beginning and maintained high parasite loads throughout the course of infection (Figure 2A, left), also assessed by area under the curve (AUC) (Figure 2A, right). Having established the importance of AHR for *Cryptosporidium* infection control, we next wanted to know which cells were playing a role in this defense. Using AHR-reporter mice expressing AHR-tdTomato, we determined that the majority of epithelial cells express AHR (Figure 2B), consistent with previous observations.^39^ Because *Cryptosporidium* only infects epithelial cells, we first challenged mice with an intestinal epithelial cell (IEC)-specific AHR KO (Vil-Cre AHR^fl/fl^ termed AHRKO^Epithelium^) with *Ct*-CR to probe whether IEC-intrinsic AHR expression influenced parasite burden. Surprisingly, we found that epithelial AHR signaling was dispensable for control of parasite growth (Figure 2C).

AHR is also expressed by immune cells in the gut (Figure 2D). We next asked whether AHR expression in immune cells is required to limit *Ct*-CR. To achieve this, we crossed hematopoietic cell-specific Vav-Cre mice with AHR^fl/fl^ mice to produce immune cell-specific AHR-deficient mice (AHRKO^Immune^). Consistently, mice with AHR deficiency in their immune cells were highly susceptible to *Ct*-CR infection compared with littermate controls (Figure 2E), even when parasite burdens were followed for a longer duration of infection (Figure S1). Vav-Cre is also expressed by endothelial cells,^40^ which express high levels of AHR.^39^ Therefore, AHRKO^Immune^ mice could simultaneously delete AHR in both their immune and endothelial cells. To account for a potential role for AHR in anti-cryptosporidial defense via the endothelium only, we made use of a tamoxifen-inducible Cre line controlled by the cadherin 5 (Cdh5) promoter to delete AHR selectively in endothelial cells. Cdh5^CreERT2^AHR^fl/fl^ (AHRKO^Endo^) and littermate control mice were administered tamoxifen orally. 5 days post-tamoxifen treatment, (AHRKO^Endo^) mice were infected with *Ct*-CR, alongside a cohort of AHRKO^Immune^ mice in the same experiment. In endothelial cell-specific AHR KO mice, *C. tyzzeri* levels were comparable to those in littermate controls, whereas the infection burden was again increased in AHRKO^Immune^ mice (Figure 2F). *C. tyzzeri* levels in the ileum of AHRKO^Immune^ mice were also similar to the levels in total-body-AHR-deficient mice (AHRKO^Body^) (Figure 2G). Hence, these results narrowed down the importance of AHR expression specifically in immune cells to control an intestinal *C. tyzzeri* infection. We also noted that the increased parasite burden in AHRKO^Immune^ mice was associated with increased expression of enterocyte markers at the expense of stem cell and Tuft cell markers (Figure 2H). Taken together, AHR signaling in immune cells is vital to regulate the ability of *Cryptosporidium* to grow in intestinal epithelial cells of the small intestine.

### AHR-expressing CD8a IELs respond to *C. tyzzeri* parasite infection

**Figure 3:**
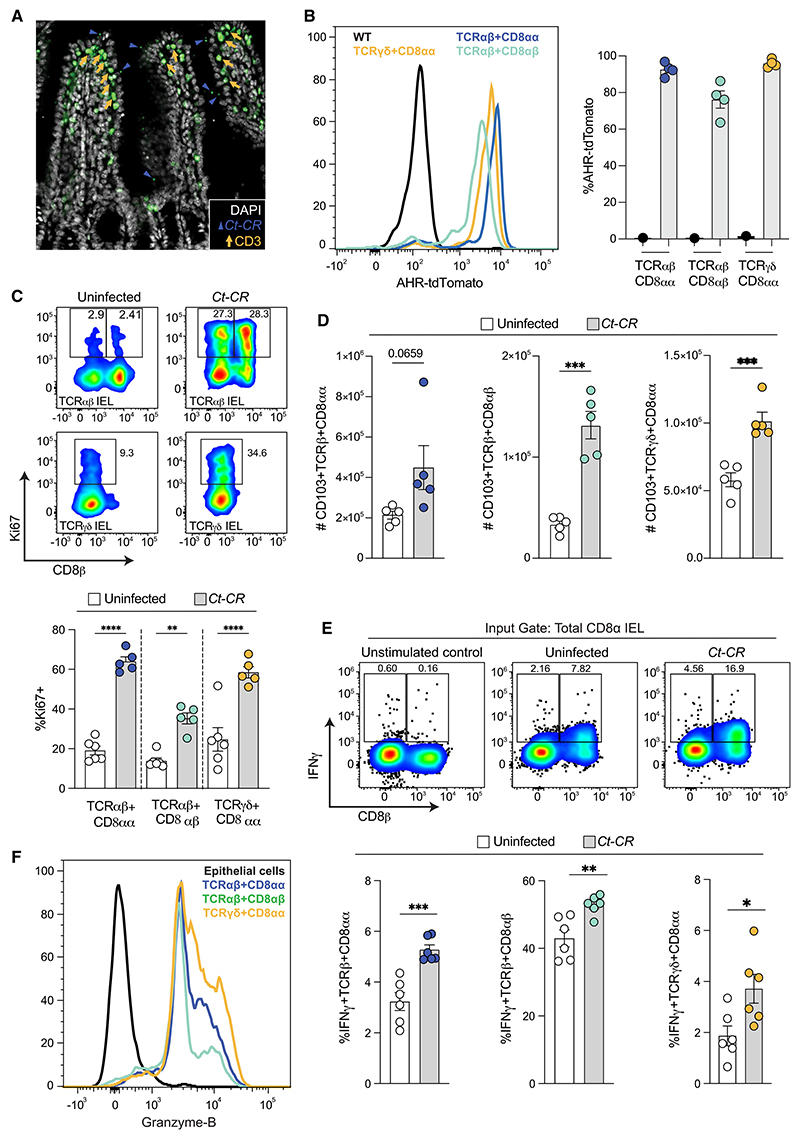
AHR-expressing CD8a IELs respond to *C. tyzzeri* parasite infection (A) Representative image showing *Ct-*CR (navy blue arrowhead pointing to mNeonGreen-positive parasites on the luminal side of villi) and the close proximity of IELs (orange arrow pointing to CD3^+^ IELs) to infected epithelial cells in the ileum. (B) AHR-tdTomato expression by TCRαβ and TCRγ δ CD8^+^ IEL subsets. Representative histogram (left) and percentage AHR-tdTomato-positive IEL sub-sets (right). (C) Ki67^+^ proliferating IELs in *Ct-*CR-infected WT mice. Representative fluorescence-activated cell sorting (FACS) plots (top) and percentage Ki67-positive IEL subsets (bottom). (D) Number of IELs in the small intestine of *Ct-*CR-infected WT mice. (E) Intracellular IFNγ levels of TCRαβ and TCRγ δ CD8^+^ IEL subsets. Representative FACS plots showing IFNγ expressing CD8αβ^+^ and CD8αα^+^ IELs (top) and percentage IFNγ ^+^TCRαβ and TCRγ δ CD8^+^ IEL subsets (bottom) in naive and infected mice. (F) Histogram of intracellular granzyme-B in TCRαβand TCRγ δCD8^+^ IEL subsets. CD45-negative epithelial cells are used as negative controls. (A–F) Representative results of at least 2 independent experiments. Each dot represents individual mice. Error bars, mean + SEM. *p < 0.05, **p < 0.01, ***p < 0.001, ****p < 0.0001 as calculated by t test or one-way ANOVA with Tukey post-test.

Lymphocytes in the small intestine are spatially organized into intraepithelial and lamina propria layers. The intraepithelial layer is enriched with cytotoxic CD8^+^ IELs, which are the primary responders to epithelial damage due to their close proximity to epithelial cells.^23^ Immunofluorescence images of mouse ileal sections show CD3^+^ IELs in close contact with *C. tyzzeri-*infected epithelial cells (Figure 3A). Using AHR-tdTomato reporter mice, we found that all the major IEL subsets, including TCRαβ^+^, CD8αα ^+^, TCRαβ^+^, CD8αβ^+^andTCRγδ^+^, CD8αα^+^, express AHR-tdTomato (Figures 3B and S2). TCRαβ^+^ and TCRγδ^+^IELs from *Ct*-CR-infected mice had increased expression of Ki67, indicative of a hyperproliferative state (Figure 3C), which corresponded with increased numbers of IELs in *Ct-*CR-infected mice (Figure 3D). A closer examination of the small intestine revealed a robust increase in all three IEL subtypes, specifically in the jejunum and ileum, during the peak of infection, whereas only modest increases of TCRαβ^+^,CD8αβ^+^ and TCRγδ^+^, CD8αα^+^were seen in the duodenum (Figure S3). Moreover, IFNγ -expressing CD8^+^ IELs were significantly increased following *Ct-*CR infection (Figure 3E). CD8^+^ IELs are quiescent at steady state with minimal proliferation (Figure 3C); however, they rapidly mount a cytotoxic response to kill target cells through effector proteins such as granzymes.^41^ Indeed, all three major types of CD8^+^ IELs express granzyme-B at steady state (Figure 3F). Therefore, IELs sense epithelial invasion by *C. tyzzeri* in a similar manner to other infection settings^25,41^ and respond by producing IFNγ and granzyme-B, which are effector mediators of IEL cytotoxicity.

### AHR-dependent CD8a IELs confer resistance to *C. tyzzeri* infection in immunodeficient mice

AHR expression and signaling are essential for both TCRαβ^+^ and TCRγδ^+^ IEL survival, maintenance, and effector function.^29,42,43^

**Figure 4:**
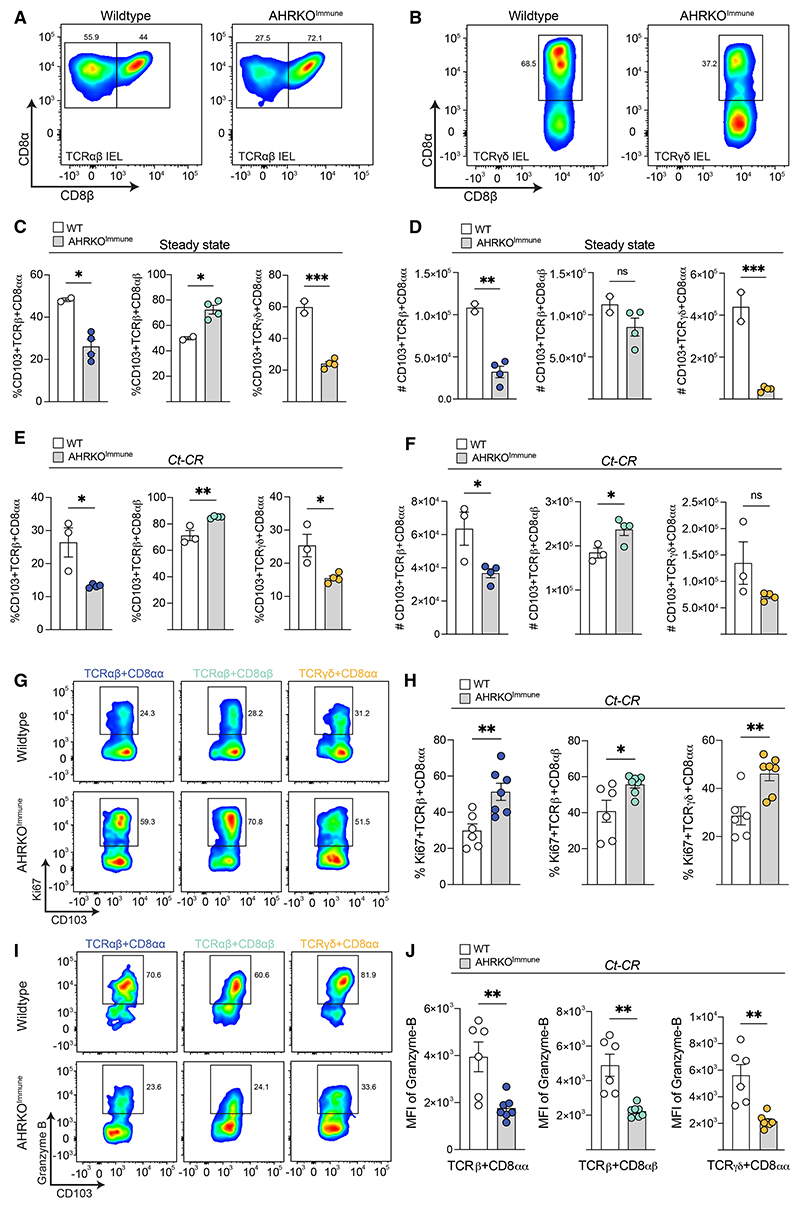
AHR expression influences the CD8a IEL response to *C. tyzzeri* infection (A and B) Representative FACS plots showing the percentage of TCRαα^+^ CD8αα, TCRαα^+^ CD8αβ IEL subsets (A) and TCRβγ^+^ CD8αβ(B) in WT and AHRKO^Immune^ mice at steady state.

In line with these findings, the percentage and total numbers of natural CD8αα IELs recovered from naive AHRKO^Immune^ mice were significantly lower compared with WT littermates, whereas the numbers of TCRαβ^+^ CD8αβ^+^ IELs were similar (Figures 4A–4D). During infection with *Ct-*CR, TCRαβ^+^ CD8αβ^+^ and TCRγδ^+^ CD8αα^+^ IELs were similarly reduced, but TCRαβ^+^ CD8αβ^+^ IELs increased in AHRKO^Immune^ mice (Figures 4E and 4F). AHR-deficient CD8_α_ IELs were hyper-proliferative (Figures 4G and 4H). However, they exhibited diminished cytolytic activity indicated by decreased granzyme-B levels (Figures 4I and 4J). Therefore, it is likely that a decrease in cytotoxic CD8^+^ IELs in AHR-deficient mice contributed to the increased *Ct*-CR burden.

**Figure 5:**
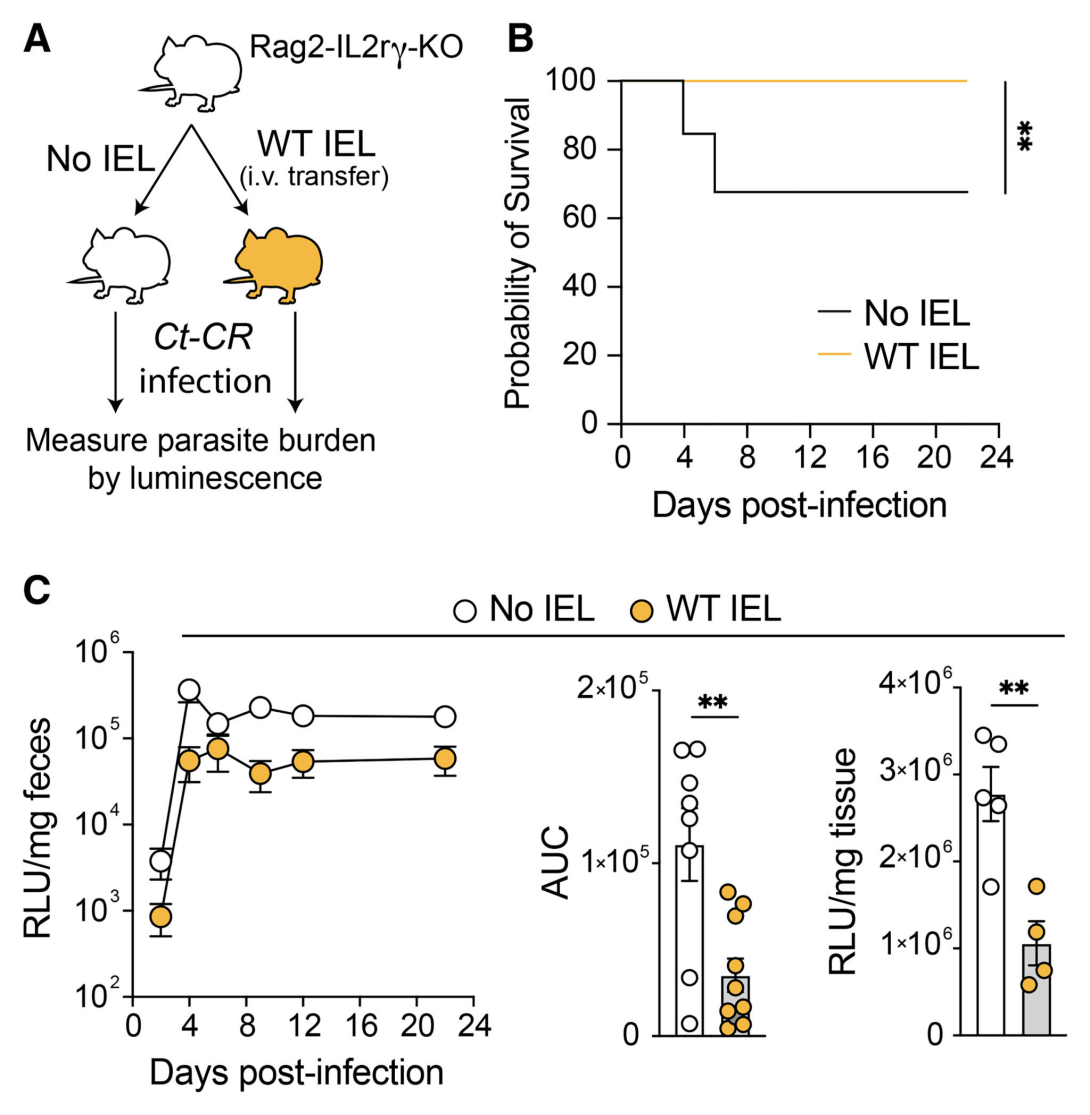
CD8a IELs efficiently control *C. tyzzeri* parasite growth in immunodeficient mice (A) Experimental design of Rag2-IL2Rγ-CD47 triple knockout mice receiving CD8^+^ IELs followed by infection with *Ct*-CR. (B) Survival of Rag2-IL2Rγ-CD47 triple knockout mice infected with *Ct*-CR that received WT CD8^+^ IELs compared with controls. (C) *Ct*-CR parasite burden in the fecal samples of CD8^+^ IEL-transferred Rag2-IL2Rγ-CD47 triple knockout mice (left). The area under the curve (AUC) is pooled data from 2 independent experiments (center). *Ct*-CR levels in the ileum on DPI-22 (right); results are from one experiment. Each dot represents individual mice. Error bars, mean + SEM. **p < 0.01, as calculated by t test. (C and D) Percentage (C) and absolute number (D) of TCRαβand TCRγ δ CD8^+^ IEL subsets in AHRKO^Immune^ mice and littermate WT controls at steady state. (E and F) Percentage (E) and absolute number (F) of CD8^+^ IEL subsets on day 7 post-*Ct-*CR infection. (G and H) Representative FACS plots (G) and percentage (H) of Ki67-positive CD8α IEL subsets in *Ct-*CR-infected mice. (I) Percentage of granzyme-B-expressing CD8α IEL subsets. (J) Mean fluorescence intensity (MFI) of intracellular granzyme-B in TCRαβ and TCRγ δ CD8^+^ IEL subsets in AHRKO^Immune^ mice and littermate WT controls infected with *Ct-*CR. (A–J) Representative results of at least 2 independent experiments. Each dot represents individual mice. Error bars, mean + SEM. ns, not significant, *p < 0.05, **p < 0.01, ***p < 0.001, as calculated by t test.

To directly assess the protective function of CD8^+^ IELs, we purified CD8^+^ IELs from the small intestine of naive WT mice and transferred them intravenously into immunodeficient Rag2-IL2Rγ-CD47 triple KO mice, which are usually highly susceptible to infections.^35^ 4 weeks after IEL transfer, the mice were infected with *Ct*-CR (Figure 5A). Although untreated immunodeficient mice experienced increased severity with significant reduction in survival (Figure 5B), triple KO mice that received WT CD8^+^ IELs survived and had reduced parasite burdens in the feces (Figure 5C) and ileum (Figure 5C, right). To determine the contribution of specific IEL subsets to protection from infection, we sort-purified CD8^+^ IELs into CD8αα and CD8αβ subsets and transferred them into triple KO mice as before. Mice that received CD8αα^+^ IELs showed lower fecal and ileal parasite burdens over a long-term infection (Figure S4). In conclusion, AHR expression in CD8^+^ IELs is required for their maintenance and cytotoxicity, and these CD8^+^ IELs can efficiently control *Ct-*CR parasite growth *in vivo*.

### Dietary AHR ligands confer resistance to *C. tyzzeri* infection

**Figure 6:**
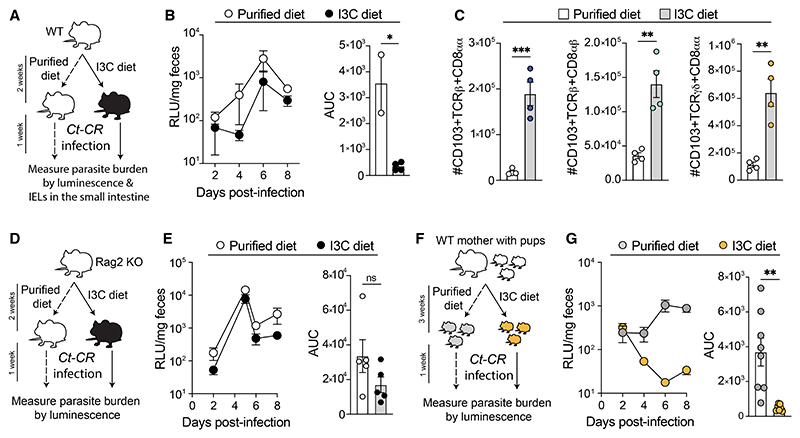
Dietary AHR ligands increase CD8α IELs, which confer resistance to *C. tyzzeri* infection (A–C) 3-to-4-week-old WT mice were fed with either purified diet or I3C diet for 2 weeks followed by infection with *Ct*-CR and maintained on the same diets (A). *Ct*-CR levels in the feces (B) and absolute number of small intestinal CD8α IELs (C) were enumerated. (D and E) Rag2 knockout mice were fed with either purified or I3C diet for 2 weeks and infected with *Ct*-CR while maintained on the same diets (D). Nanoluciferase readings indicating *Ct*-CR burden (E) monitored in the feces (left) and AUC (right). (E) Experimental design of nursing WT mothers fed with either purified or I3C diets. Their pups were weaned on the same diets and infected with *Ct*-CR. (F) *Ct*-CR parasite burden in young pups of dams that received either purified or I3C diets. (A–G) Representative results of at least 2 independent experiments. Each dot represents individual mice. Error bars, mean + SEM. ns, not significant, *p < 0.05, **p < 0.01, as calculated by t test.

AHR is a ligand-activated transcription factor. In the gut, a major source of these ligands is dietary tryptophan-derived phytochemicals and tryptophan metabolites produced by the microbiota.^44^ Because AHR expressing CD8^+^ IELs are key to anticryptosporidial activity, we wanted to determine if this function is influenced by AHR-ligand availability. The phytochemical indole-3-carbinol (I3C), enriched in cruciferous vegetables, is an AHR pro-ligand that is converted to high-affinity AHR ligands upon exposure to stomach acid.^45^ We first asked whether dietary supplementation with I3C influences CD8^+^ IELs. 3-week-old WT mice were fed either control phytochemical-free synthetic AIN93M diet (“purified control diet”) or I3C (1,000 mg/kg) enriched diet (“I3C diet”) for 2 weeks and infected with *Ct-*CR while they continued on the same diets (Figure 6A). I3C dietfed mice had reduced parasite burdens compared with the mice fed the control diet (Figure 6B). Enumeration of CD8^+^ IELs in these mice indicated that I3C diet supplementation robustly increased all subsets of CD8^+^ IELs in the small intestine (Figure 6C). To demonstrate that I3C diet-mediated protection from *Ct-*CR infection is T cell dependent, we fed Rag2 KO mice with purified control or I3C diet for 2 weeks. Infection with *Ct-*CR resulted in a similar burden of parasites in both groups of mice independent of exposure to I3C diet (Figures 6D and 6E), indicating that the protective role of I3C is abrogated in T cell-deficient mice. We then investigated the significance of an I3C-enhanced diet in ameliorating *C. tyzzeri* infection in a mouse model that recapitulates early childhood infection in humans. Because AHR signaling is known to impact fertility,^46^ pregnant WT females were maintained on normal chow diet until they gave birth. We then changed the diet of WT dams from normal chow to either a purified control diet or an I3C-enriched diet. 3 weeks later, pups were weaned on the same diet that their mothers had received and were infected with *Ct-*CR (Figure 6F). WT pups that grew up on the purified control diet were far more susceptible to infection than WT pups that grew up on the I3C diet (Figure 6G). Thus, prior exposure to I3C protects mice from *Cryptosporidium* infection.

Taken together, these data underscore the potential of dietary AHR ligands to modulate cytotoxic immune defense against *C. tyzzeri*, leading to improved elimination of infected epithelial cells and thus offering a therapeutic avenue to prevent or treat cryptosporidiosis by enhancing immunity to *Cryptosporidium* through dietary interventions.

## Discussion

Although ubiquitously prevalent, *Cryptosporidium* is particularly problematic in resource-poor settings. Malnutrition is endemic to those same settings, which is, in turn, a risk factor for *Cryptosporidium* infections and chronic diarrhea.^47^ Malnutrition and immunodeficiency increase the risk of recurrent *Cryptosporidium* infections, which contributes to increased morbidity and mortality.^48^ The findings presented in this study using a mouse model of infection offer a proof of concept for the potential use of dietary AHR ligands, such as I3C, to curb this vicious cycle of chronic infections, diarrhea, and malnutrition. Many resource-poor regions across the world already rely on ready-made food formulations, such as ready-to-use therapeutic food (RUTF), to treat children suffering from severe wastage and malnutrition, often as the result of infections, with varying degrees of success.^49^ Several human clinical trials show that I3C is safe for human consumption in adults.^50–52^ I3C supplements are commercially available on the market; thus, it is conceivable for them to be included in RUTF formulations following controlled human challenge trials confirming their therapeutic potential. Intriguingly, it has also been shown that nursing mice can pass on AHR ligands to their newborn.^53^ Therefore, there is also scope for dietary prophylactic interventions to be made at the level of nursing mothers, especially because *Cryptosporidium* infections are most severe in children less than 1 year old.

In laboratory settings, younger mice are also more susceptible to *Cryptosporidium* infections. Here, we have shown the importance of cytotoxic CD8^+^ IELs in controlling this infection in an AHR-dependent manner. Due to their close association with intestinal epithelial cells, IELs are known to be primary responders to several invading pathogens in the gut,^25,54,55^ and our findings support these observations in the context of *Cryptosporidium* infection. CD8^+^ IELs (natural CD8αα^+^ and induced CD8αβ^+^) in the gut are long-lived tissue-resident memory cells. Natural CD8αα^+^ cells populate the relatively abiotic guts of pups in response to self-antigens during weaning,^56^ whereas CD8αβ^+^ IELs accumulate over time in response to externally derived antigens.^23^ Our IEL transfer experiment separating CD8αβ^+^ and CD8αα^+^ subtypes suggests that the AHR-dependent protective effect mediated by CD8^+^ IELs is restricted to CD8αα^+^ IELs (TCRαβ^+^ CD8αα IELs and TCRγδ^+^ CD8αα). This is in line with our observation that CD8αα^+^ IELs have a slightly higher expression of AHR and are more dependent on AHR ligands for their maintenance compared with CD8αβ^+^ IELs, although cytotoxic function is equally impaired in all IELs in the absence of AHR. Furthermore, CD8αβ^+^ IELs proliferate in response to *C. tyzzeri* infection more robustly compared with CD8αα^+^ IELs. Although the exact mechanism by which these IELs sense epithelial injury during infection is unclear,^25^ being in a constant state of heightened activation with increased cytolytic granzyme expression^41^ increases their ability to swiftly act on *Cryptosporidium*-infected epithelial cells. The presence of IELs at the coalface of the mucosal barrier and their dependence on AHR^29,42,43^ opens up the possibility to modulate these IELs during *Cryptosporidium* infections through supplementation of dietary AHR ligands. Recent data also suggest that certain AHR ligands may be able to suppress infection of *C. parvum* in epithelial cells through a separate AHR-independent mechanism.^57^ Although we did not see I3C-mediated protection against *C. tyzzeri* in the absence of immune cells, it remains possible that other dietary AHR ligands may be capable of acting against the parasite through multiple cell types.

IFNγ is a key cytokine responsible for early protection from *C. tyzzeri* infection.^14,35^ Interestingly, natural CD8αα IELs do not produce as much IFNγ as their induced CD8αβ counter-parts, although both robustly respond to *C. tyzzeri* infection. However, both TCR αβ^+^ and TCR γδ^+^ IELs expressed cytotoxic granular protein granzyme-B in their quiescent and activated states. A heightened state of activation with increased cytotoxic potential and rapid proliferative capacity could facilitate swift killing of *C. tyzzeri-*infected epithelial cells by IELs. Indeed, AHR-deficient mice (AHRKO^Body^ and AHRKO^Immune^) that lack these IELs have elevated parasite burdens from DPI-2 onward, suggesting that in the absence of cytotoxic CD8^+^ IELs *C. tyzzeri* robustly establishes an infection in the small intestine, which cannot be cleared even after 4 weeks post infection. In contrast to CD8αα IELs, induced CD8αβIELs can form antigen-specific long-lived memory cells.^58^ Future studies should explore the antigen-specificity of conventional TCR αβ^+^ CD8αβ IELs during primary and secondary infections and narrow down their role in long-term protection against subsequent *Cryptosporidium* infections.

We have previously established *C. tyzzeri* as a model of *Cryptosporidium* infection to study the relationships between a pathogen and its natural host.^14^ Our work showed infection by strain *Ct*-UGA55, isolated from the *Mus musculus domesticus* mouse subspecies, caused characteristic villus blunting, a decrease in villus-crypt height ratios, and an increase in mitotic events in the epithelium of the small intestine.^14^ Here, we have furthered these studies using a *C. tyzzeri* strain obtained from an Eastern European mouse subspecies (*M. m. musculus*), which we found to produce many hallmarks of epithelial damage. There is increased expression of *Ifng* at the tissue level, a loss of markers of differentiated cells, such as *Chga* and *Dclk1*, as well as an increase in the enterocyte marker *Car4*. Most notably, Sca-1 protein levels significantly increased in infected epithelial tissue, a notable marker of epithelial damage previously seen in mouse models of colitis.^37,38^ Increased Sca-1 expression is also caused during infection by the intestinal parasitic helminth *H. polygyrus* in an IFNγ -dependent manner and is indicative of a reversion to a fetal-like state in tissue.^59^ We hope that this study paves the way for future work understanding how this parasite specifically damages the small intestine, potentially triggering a re-wiring of the epithelial regenerative response.

Taken together, we have further uncovered host responses to *C. tyzzeri* and revealed the role of environmental sensor AHR and its natural ligands in conferring protection from *Cryptosporidium* infection by modulating gut-resident cytotoxic lymphocytes using a well-defined genetically tractable mouse model. This study extends our understanding of AHR signaling and its importance in maintaining gut barrier integrity in the small intestine and suggests a way forward toward future therapeutic strategies to control the severity of *Cryptosporidium* infections.

## Star+Methods

Key Resources Table

**Table:** 

REAGENT or RESOURCE	SOURCE	IDENTIFIER
Antibodies		
Anti-mouse Granzyme-B	Biolegend	Cat# 515408; Clone: GB11; RRID: AB_2562196
Anti-mouse TCRβ	Biolegend	Cat# 109226; Clone: H57-597; RRID: AB_1027649
Anti-mouse CD4	Biolegend	Cat# 100557; Clone: RM4-5; RRID: AB_2562607
Anti-mouse CD8β	BD Biosciences	Cat# 740952; Clone: H35-17.2; RRID: AB_2740577
Anti-mouse TCRγδ	Biolegend	Cat# 118106; Clone: GL3; RRID: AB_313830
Anti-mouse IFNγ	BD Biosciences	Cat# 557724; Clone: XMG1.2; RRID: AB_396832
Anti-mouse CD8α	Biolegend	Cat# 100734; Clone: 53-6.7; RRID: AB_2075238
Anti-mouse CD103	eBiosciences	Cat# 12-1031-82; Clone: 2E7; RRID: AB_465799
Anti-mouse CD45	Biolegend	Cat# 103146; Clone: 30-F11; RRID: AB_2564003
Anti-mouse TCRγδ	Biolegend	Cat# 118123; Clone: GL3; RRID: AB_11203530
Anti-mouse Ki67	eBiosciences	Cat# 17-5698-82; Clone: SolA15; RRID: AB_2688057
Anti-mouse TCRβ	Biolegend	Cat# 109224; Clone: H57-597; RRID: AB_1027648
Anti-mouse CD3	Biolegend	Cat# 100210; Clone: 17A2; RRID: AB_389301
Anti-mouse Sca-1	Biolegend	Cat# 122501; Clone: E13-161.7; RRID: AB_756186
Anti-mouse EpCAM	Biolegend	Cat# 118222; Clone: G8.8; RRID: AB_2563322
Bacterial and virus strains		
Stable competent *E. coli*	NEB	Cat# C3040H
Chemicals, peptides, and recombinant proteins		
Taqman 2X universal PCR master mix	ThermoFisher Scientific	Cat# 4318157
TRI reagent Solution	Invitrogen	Cat# 9738G
Percoll	Amersham	Cat# 17-0891-01
DAPI	Sigma	Cat# D9542
PMA	Sigma	Cat# P1585
Ionomycin	Sigma	Cat# I0634
Brefeldin-A	Biolegend	Cat# 420601
Critical commercial assays		
eBioscience FOXP3/Transcription factor staining buffer set	Invitrogen	Cat# 00-5523-00
LIVE/DEAD Fixable Near-IR Dead Cell Stain Kit	Invitrogen	Cat# L10119
NanoGlo Luciferase kit	Promega	Cat# N1150
CountBright Absolute Counting Beads	Invitrogen	Cat# C36950
EasySep™ Mouse CD8a Positive Selection Kit II	StemCell Technologies	Cat# 18953
cDNA Reverse Transcription Kit	Thermofisher	Cat# 4368814
Deposited data		
Whole genome sequencing data of *C. tyzzeri-CR*	This study	NCBI BioProject PRJNA950368
Experimental models: Organisms/strains		
C57BL/6J mice	The Jackson Laboratory	Strain# :000664; RRID: IMSR_JAX:000664
IFNg knockout mice	The Jackson Laboratory	Strain# :002287; RRID: IMSR_JAX:002287
AHR-tdTomato mice	Diny et al.^39^	N/A
AHR knockout mice (AHRKO^Body^)	The Jackson Laboratory	Strain #:002831; RRID: IMSR_JAX:002831
Vil-Cre AHR^fl/fl^ (AHRKO^Epithelium^) mice	Shah et al.^38^	N/A
Vav-Cre AHR^fl/fl^ (AHRKO^Immune^) mice	This study	N/A
Cdh5^CreERT2^AHR^fl/fl^ (AHRKO^Endo^) mice	This study	N/A
Rag2-IL2Rγ-CD47 knockout mice	The Jackson Laboratory	Strain#: 025730; RRID: IMSR_JAX:025730
*C. tyzzeri-CR* parasites	Kváč et al.^33^	*Cryptosporidium tyzzeri* CR2206
Oligonucleotides		
IFNg	TaqMan™ Assays	Cat# Mm01168134_m1
Olfm4	TaqMan™ Assays	Cat# Mm01320260_m1
Clu	TaqMan™ Assays	Cat# Mm01197004_m1
Sox9	TaqMan™ Assays	Cat# Mm00448840_m1
Chga	TaqMan™ Assays	Cat# Mm00514341_m1
Dclk1	TaqMan™ Assays	Cat# Mm00444950_m1
Muc2	TaqMan™ Assays	Cat# Mm01276696_m1
Car4	TaqMan™ Assays	Cat# Mm00483021_m1
HPRT	TaqMan™ Assays	Cat# Mm00446968_m1
B2M	TaqMan™ Assays	Cat# Mm00437762_m1
Recombinant DNA		
Cas9 expression vector with *C. tyzzeri* thymidine kinase locus guide	Sateriale et al.^14^	N/A
*Cryptosporidium* Nanoluciferase-Neomycin^R^-mNeonGreen repair template	This paper	N/A
Software and algorithms		
Flowjo	Tree Star	https://www.flowjo.com/
Prism (Version: 9)	Graphpad	https://www.graphpad.com/
Endnote	Endnote	https://endnote.com/
QuPath	GitHub	https://qupath.github.io/
FIJI (version 2.1.0/1.53c)	Schindelin et al.^60^	https://fiji.sc/
Burrows-Wheeler Aligner	Sourceforge	https://bio-bwa.sourceforge.net/
Samtools	GitHub	https://github.com/samtools/
GATK	Broad Institute	https://gatk.broadinstitute.org/hc/en-us
SnpEff	GitHub	http://pcingola.github.io/SnpEff/
Circlize package for R	CRAN	https://cran.r-project.org/web/packages/circlize/index.html
Other		
Purified diet (AIN93M)	ssniff	Cat# E15713-047
I3C diet (AIN93M+1000mg/kg indole-3-carbinol)	ssniff	Cat# S9477-E724

### Resource Availability

#### Lead contact

For further information and access to parasites, reagents, or mice used in the study please address the lead contact, Adam Sateriale (adam.sateriale@crick.ac.uk).

#### Materials availability

Parasite lines generated in this study and vectors used to generate parasite lines are available on request.

### Experimental Model Details

#### Mice

Mice used in this study including C57BL/6J, IFNγ knockout, AHR-tdTomato, AHR-deficient (AHRKO^Body^), AHR^fl/fl^, Villin-Cre, Vav-Cre, CDH5-CreERT2, Rag2 knockout, and Rag2-IL-2rγ-CD47 triple knockout mice were bred in the Francis Crick Institute Biological Research Facility under specific pathogen-free conditions. Vil-Cre AHR^fl/fl^ (AHRKO^Epithelium^) mice were made by crossing Villin-Cre (Jackson Laboratory; RRID: IMSR_JAX:004586) with AHR^fl/fl^ mice, created in-house from *Ahr*^*tm1a(KOMP)Mbp*^ ES cells. Vav-Cre AHR^fl/fl^ (AHRKO^Immune^) mice were made by crossing Vav-Cre (Jackson Laboratory; RRID: IMSR_JAX:008610) with AHR^fl/fl^ mice. B6.Cdh5(PAC)-CreERT2 mice were imported from Taconic on a C57BL/6J background (originally developed in the laboratory of Ralf Adams at the London Research Institute, CRUK). These were intercrossed with AHR^fl/fl^ mice to create the Cdh5^CreERT2^AHR^fl/fl^ (AHRKO^Endo^) mouse line. Mouse experiments were conducted as per the guidelines detailed in the Project Licences granted by the UK Home Office to Brigitta Stockinger (PP0858308) and Adam Sateriale (PP8575470).

#### Parasite strains

The *C. tyzzeri*-CR strain was obtained from Martin Kváč (Biology Centre of the Czech Academy of Sciences). Using this, the *Ct-CR* mNeonGreen parasite line was created for this study by Cas9-directed homology repair using a template carrying genes expressing Nanoluciferase and Neomycin resistance as described in the STAR Methods section below.

### Method Details

#### Mouse infections

Both male and female mice aged between 3-6 weeks old were randomly assigned to experimental groups. No statistical tests were performed to pre-determine sample sizes for experimental groups; rather, they were determined by available litter sizes. No animals were excluded from experimental results. For infection experiments, 50,000 *Ct-*CR oocysts were administered to each mouse by oral gavage. Parasite burden was measured in the feces and intestinal tissues.

#### Genome sequencing and alignment

Genomic DNA was isolated from 2×10^7^ *C. tyzzeri* sporozoites using a Qiagen DNeasy Blood & Tissue Kit. A DNA library was prepared with a Nextera XT DNA Library Preparation Kit (Illumina) and 150bp paired end reads were obtained on a MiSeq patform (Illumina). To align the reads, the refence genome for *C. tyzzeri* (UGA55) was first downloaded from CryptoDB.^61^ Reads were aligned using Burrows-Wheeler Aligner (bwa mem) and converted into bam format using Samtools.^62^ Duplicate reads were then removed and re-indexed using GATK. Variants were called with GATK HaplotypeCaller using a ploidy of 1 (*Cryptosporidium* sporozoites are haploid).^63^ Variants were finally annotated with SnpEff^64^ and plotted against the reference genome using the circlize package for R.^65^

#### Generation of transgenic parasites

Transgenic *C. tyzzeri*-CR parasites were created using methods described previously.^66,67^ Briefly, 1.5 × 10^6^ oocysts were bleach-treated and incubated at 37 °C in 0.75% sodium taurocholate for 1 h to promote excystation. Sporozoites were transfected with a plasmid expressing Cas9 and guide targeting the *C. tyzzeri* TK gene, along with a repair template carrying Nanoluciferase, mNeon-Green, and Neomycin resistance genes. IFNγ ^-/-^ mice were orally gavaged with these transfected sporozoites, following a sodium bicarbonate oral gavage a few minutes prior. Mice were given Paromomycin in their drinking water to select for transgenic parasites and Nanoluciferase levels in fecal samples were tracked over time.

#### Measuring parasite burden in tissues and feces

Nanoluciferase readings from fecal samples were performed as a proxy for parasite burdens as previously described using the NanoGlo Luciferase reporter assay (NanoGlo Luciferase kit, Promega, N1150).^68^ Small intestinal tissue sections were cut, weighed, and processed in a similar manner to fecal samples.

#### Isolation of parasites

Parasites were isolated from daily mouse fecal collections by sucrose flotation followed by cesium chloride-mediated density gradient centrifugation as described previously.^67^ Briefly, 5–7 days’ worth of fecal samples were made into a slurry in tap water and filtered through a sieve of 250 um pore size. Filtrates were mixed 1:1 with a sucrose solution (1.33 specific gravity) and centrifuged at 1000 g at 4 °C. The supernatant was washed in cold water (1:100), centrifuged again at 1500 g at 4 °C and resuspended in cold saline. This suspension was carefully laid over a cesium chloride solution (1.25 M) in a 1:1 ratio and centrifuged at 16,000 g at 4°C to produce an interphase containing suspended oocysts. These were collected and stored in cold saline for future use.

#### Isolation and transfer of IELs

IELs were isolated from the small intestine. The small intestine was cut open longitudinally and washed twice in Ca^2+^ and Mg^2+^-free PBS to remove the intestinal contents. Cleaned intestine was cut into 1cm pieces and resuspended with HBSS (Ca^2+^ and Mg^2+^-free) containing 5% FCS and 2mM EDTA. Tissue pieces were incubated for 30 min at 37°C with shaking at 200 r.p.m. Single cell suspension from the epithelial wash was resuspended in 36% Percoll (Amersham) and layered on top of 67% Percoll (Amersham; Cat# 17-0891-01) and subjected to density gradient centrifugation at room temperature (700g for 30 min). Intermediate layer containing IELs was collected and washed with 1X PBS followed by either flow cytometry analysis or used for CD8α IELs purification using EasySep™ mouse CD8α positive selection kit-II (StemCell Technologies; Cat# 18953). Sort purified total CD8α IELs (50,000/mouse), or CD8αα (120,000/mouse) and CD8αβ (25,000/mouse) IELs were intravenously injected into recipient Rag2-IL-2rγ-CD47 knockout mice. Staining and gating strategies are provided below and in Figure S2. For measurement of intracellular IFNγ ex-vivo harvested IELs were restimulated with PMA (20ng/ml final concentration) and ionomycin (1μM final concentration) in the presence of brefeldin-A (10μg/ml final concentration) for 3 h. In some experiments, epithelial cells (top layer of 36% Percoll gradient) that were separated from IELs were used for RNA extraction.

#### Flow cytometry

Single cell suspension was first stained with LIVE/DEAD Near-IR dead cell stain (Invitrogen; Cat# L10119). Cell suspensions were then incubated with surface staining antibodies targeting CD45 (Cat# 103146; Clone: 30-F11), CD103 (Cat# 12-1031-82; Clone: 2E7), TCRβ (Cat# 109224; Clone:H57-597), TCRγ δ (Cat# 118123 & Cat# 118106; Clone:GL3), CD4 (Cat# 100557; Clone: RM4-5), CD8α (Cat# 100734; Clone:53.6-7), and CD8β (Cat# 740952; Clone: H35-17.2). For intracellular protein staining, cells were fixed and permeabilized using eBioscience FOXP3 staining kit (Invitrogen; Cat# 00-5523-00) followed by intracellular staining of Ki67 (eBiosciences; Cat# 17-5698-82; Clone: SOIA15), Granzyme-B (Biolegend; Cat# 515408; Clone:GB11) and IFNγ (BD Biosciences; Cat# 557724; Clone: XMG1.2) as per the experimental condition. Acquired data was analyzed using Flowjo software (https://www.flowjo.com/). Identification of different subsets of IELs is described in Figure S2.

#### Immunohistochemistry and immunofluorescence staining

Mouse ileal sections were fixed in 4% PFA for at least 4 h and then treated in a solution of 10 % glycerol and 25% sucrose prior to embedding in OCT. Sections were stained with anti-CD3-AF488 (1: 200; Cat# 100210; Clone: 17A2), EpCAM-AF594 (1:200; Cat# 118222, Clone: G8.8), and DAPI (1:1000; Cat# D9542). Some sections were stained with Sca-1 (1: 200; Cat# 122501; Clone: E13-161.7.) Images were taken with a laser scanning confocal microscope (Zeiss LSM 880) and processed using FIJI (ImageJ version 2.1.0/1.53c).

#### Bioimage analysis of Sca-1 immunofluorescence staining

OCT embedded cryosections were stained with Sca-1 as mentioned above. Quantification of Sca-1+ areas in the small intestinal epithelium was performed blindly using QuPath software.^69^ The epithelial layer of the villi in the small intestine was delineated manually using the polygon or brush tool in QuPath. 6 villi per small intestine were analyzed. Using the automated quantification tool, mean fluorescence intensity per area unit was estimated. This was repeated in sections stained without primary antibody as controls. The average fluorescence intensity values from the control sections were subtracted from the fluorescence intensity values obtained from each villi in the original samples. The fluorescence intensity values from 6 villi per small intestine were averaged, to report the mean GFP intensity per mouse.

#### Quantitative real-time polymerase chain reaction

RNA from the gut tissues and epithelial cells was extracted using TRIzol RNA purification kit (Invitrogen; Cat# 9738G). cDNA was synthesized with high-Capacity cDNA Reverse Transcription Kit (Thermofisher; Cat# 4368814) and qRT-PCR was performed using Taqman 2x universal PCR master mix (Thermofisher; Cat# 4318157) with appropriate primer sets. Data was normalized to house-keeping genes HPRT or B2M.

#### Dietary intervention

For dietary intervention studies mice were fed with synthetic purified control diet (AIN93M) (ssniff; Cat# E15713-047) or AIN93M diet supplemented with indole-3-carbinol (I3C) (1000mg/kg) (ssniff; Cat# S9477-E724) *ad libitum*.

### Quantification and Statistical Analysis

All statistical analyses were performed with GraphPad Prism software (version 9) (https://www.graphpad.com/). For comparison of 2 groups unpaired Student’s t test was used and for multiple comparison analyses a one-way ANOVA followed by Tukey’s multiple comparison test was performed. p < 0.05 was considered as significant. Error bars represent mean with standard error mean (SEM). ns, not significant, *p < 0.05, **p < 0.01, ***p < 0.001, ****p < 0.0001.

## Supplementary Material

Figure S1

Table S1

## Data Availability

Whole genome sequencing data of the *C. tyzzeri*-CR strain have been deposited under NCBI BioProject number PRJNA950368 and are publicly available as of the date of publication. Microscopy images used in this paper are available upon request. No new code was created for this study. All other raw data, including any additional information required to reanalyze the data reported in this paper, are also available on request.

## In brief

*Cryptosporidium* causes global diarrheal disease. Maradana, Marzook, et al. discover that certain intestinal immune cells, whose survival and function rely on AHR expression, play a vital role in defending against *Cryptosporidium* infection. Diet supplementation with AHR ligands enhances the anti-cryptosporidial activity of these immune cells.

## Highlights

- CD8^+^ IELs control *Cryptosporidium* infection in mice
- Anti-cryptosporidial action by CD8^+^ IELs requires AHR expression
- IEL cytotoxic activity diminishes in the absence of AHR
- IEL cytotoxic activity is augmented via AHR activation by dietary ligands
