# Supplementary material for: Dietary environmental factors shape the immune defense against Cryptosporidium infection
Source: Cell Host Microbe. Author manuscript; Available in PMC 2026 Mar 20. (PMC7618106; doi:10.1016/j.chom.2023.11.008)
Supplement: Figure S1 [file EMS208360-supplement-Figure_S1.pdf]

Cell Host & Microbe, Volume *31*

Supplemental information

Dietary environmental factors shape the immune defense against *Cryptosporidium* infection

Muralidhara Rao Maradana, N. Bishara Marzook, Oscar E. Diaz, Tapoka Mkandawire, Nicola Laura Diny, Ying Li, Anke Liebert, Kathleen Shah, Mauro Tolaini, Martin Kváč, Brigitta Stockinger, and Adam Sateriale

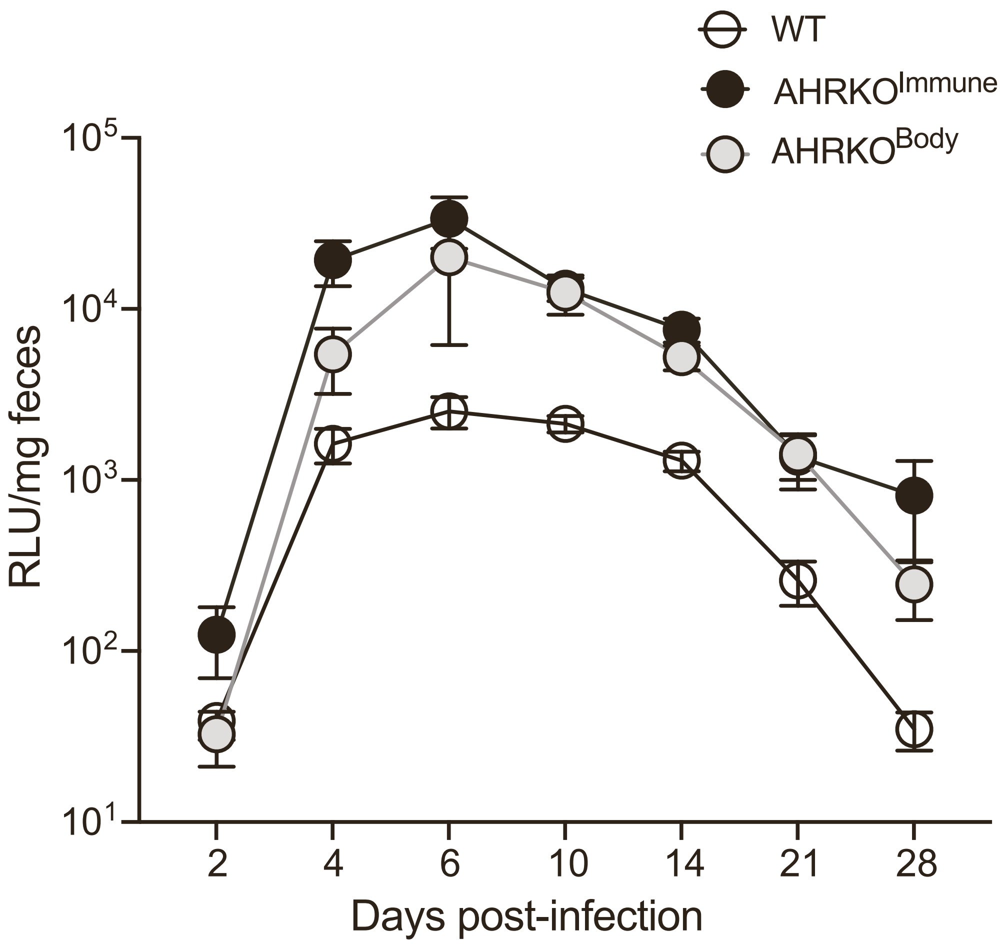

Supplementary Figure S1: Hematopoietic cell-specific loss of AHR results in higher parasite burden in the long-term (Related to Figure 2). Ct-CR parasite growth in WT (Vav^{Cre-} $AHR^{fl/fl}$), immune cell-specific AHR knockout (Vav^{Cre+} $AHR^{fl/fl}$; termed $AHRKO^{Immune}$), and total body AHR knockout ($AHRKO^{Body}$) mice monitored by Nanoluciferase readings of fecal samples over 4 weeks. Error bars, mean + SEM.

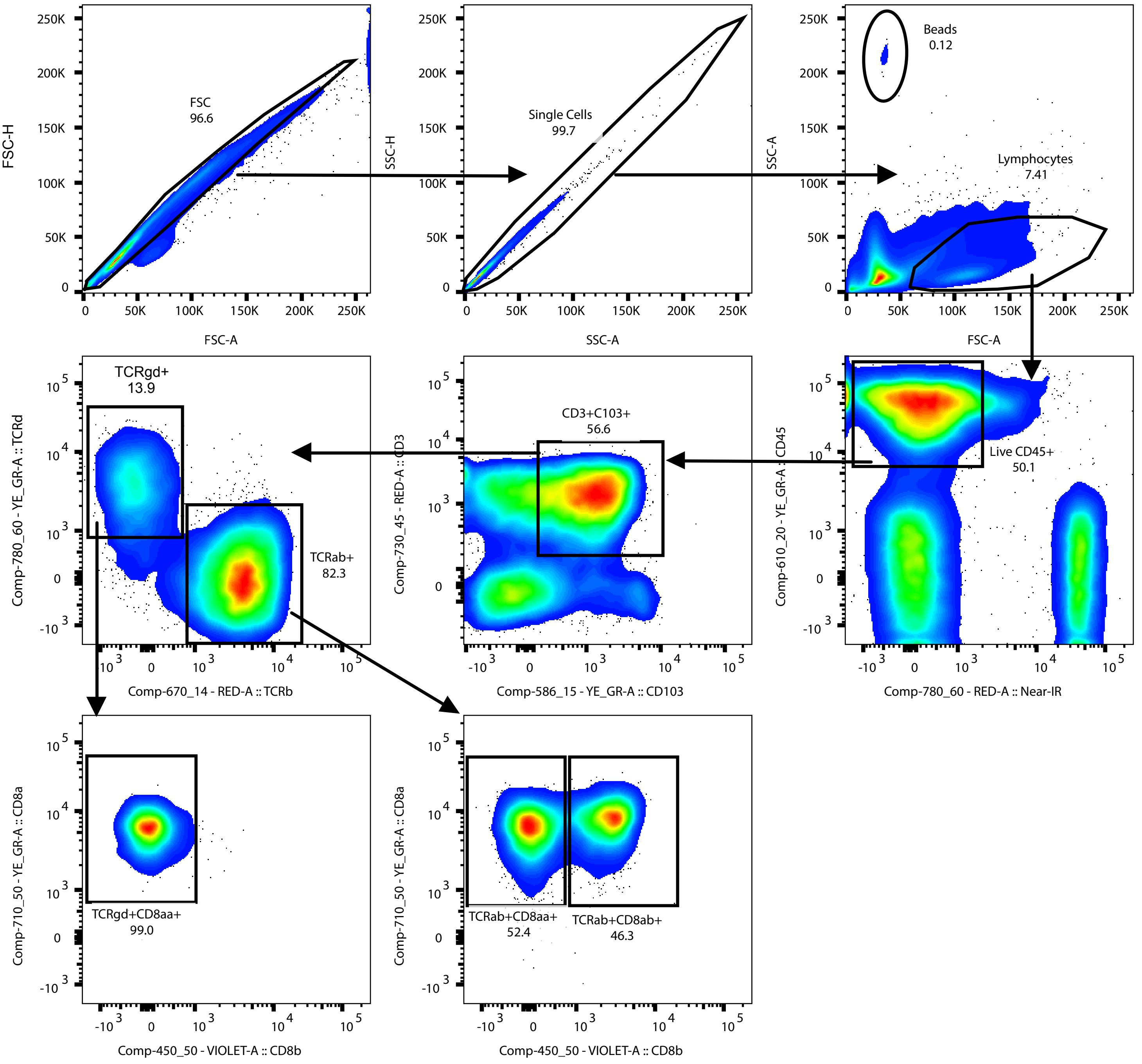

Supplementary Figure S2: Gating strategy for small intestinal intraepithelial lymphocytes (Related to Figure 3). Single cells were gated based on the forward and side scatter properties. Live CD45+ cells that were negative for Live/Dead Near-IR are used to identify CD103+ tissue resident lymphocytes. CD103+ TCRαβ+ CD8αα, CD103+ TCRαβ+ CD8αβ and CD103+ TCRγδ+ CD8αα IEL subsets were gated from CD45+CD103+ lymphocytes. CountBright Absolute Counting Beads were used to calculate the absolute number of IELs in the small intestine.

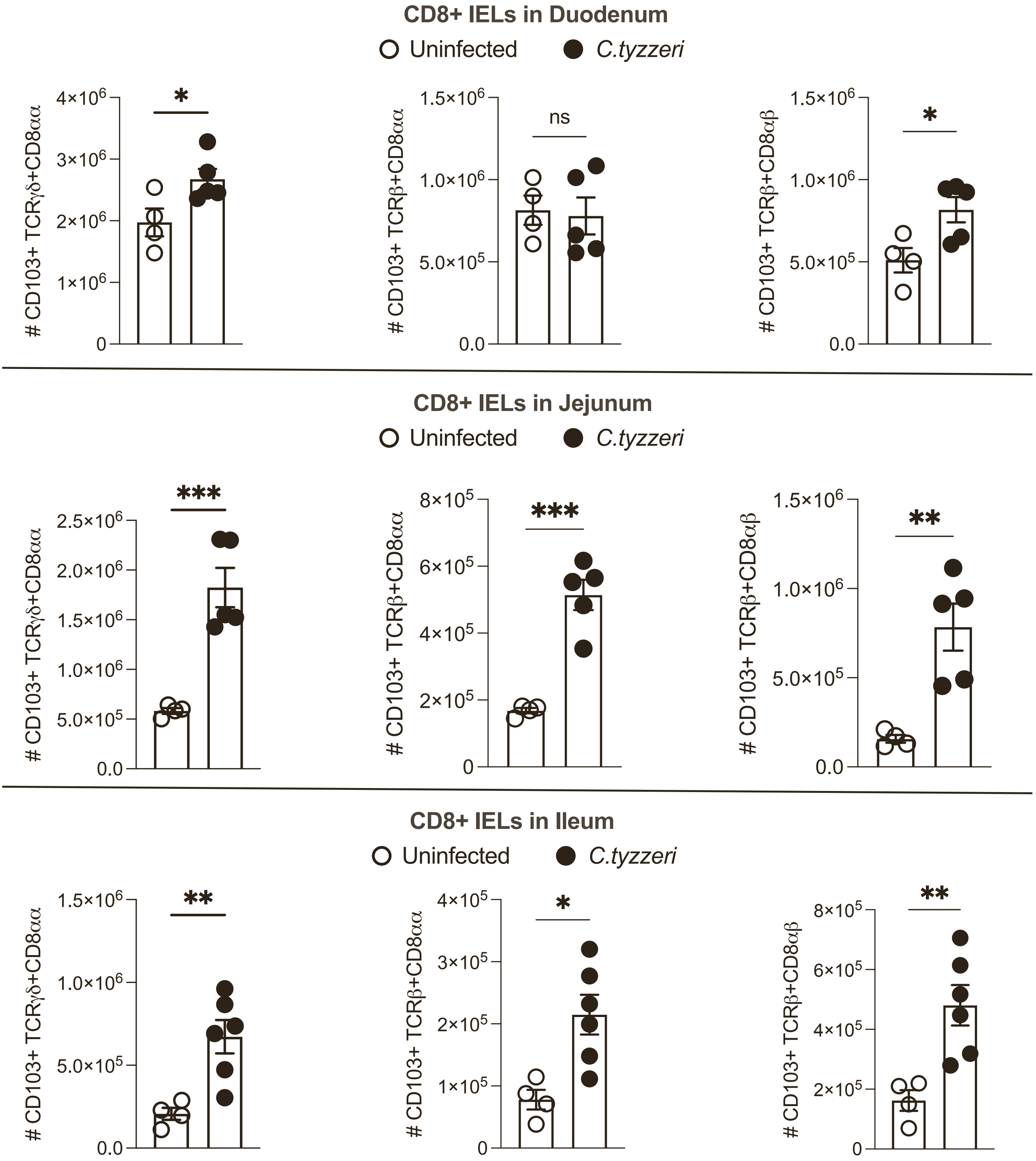

Supplementary Figure S3: CD8+ IEL numbers expand during infection across the small intestine (Related to Figure 3). Wild-type mice were infected with Ct-CR and were culled on day 6 post-infection along with uninfected age-matched controls. Small intestinal samples were taken and divided into duodenal, jejunal and ileal sections. IELs from each section were enumerated by flow cytometry. Each dot represents individual mice. Error bars, mean + SEM. ns- not significant, $*p < 0.05$, $**p < 0.01$, $***p < 0.001$, as calculated by t-test.

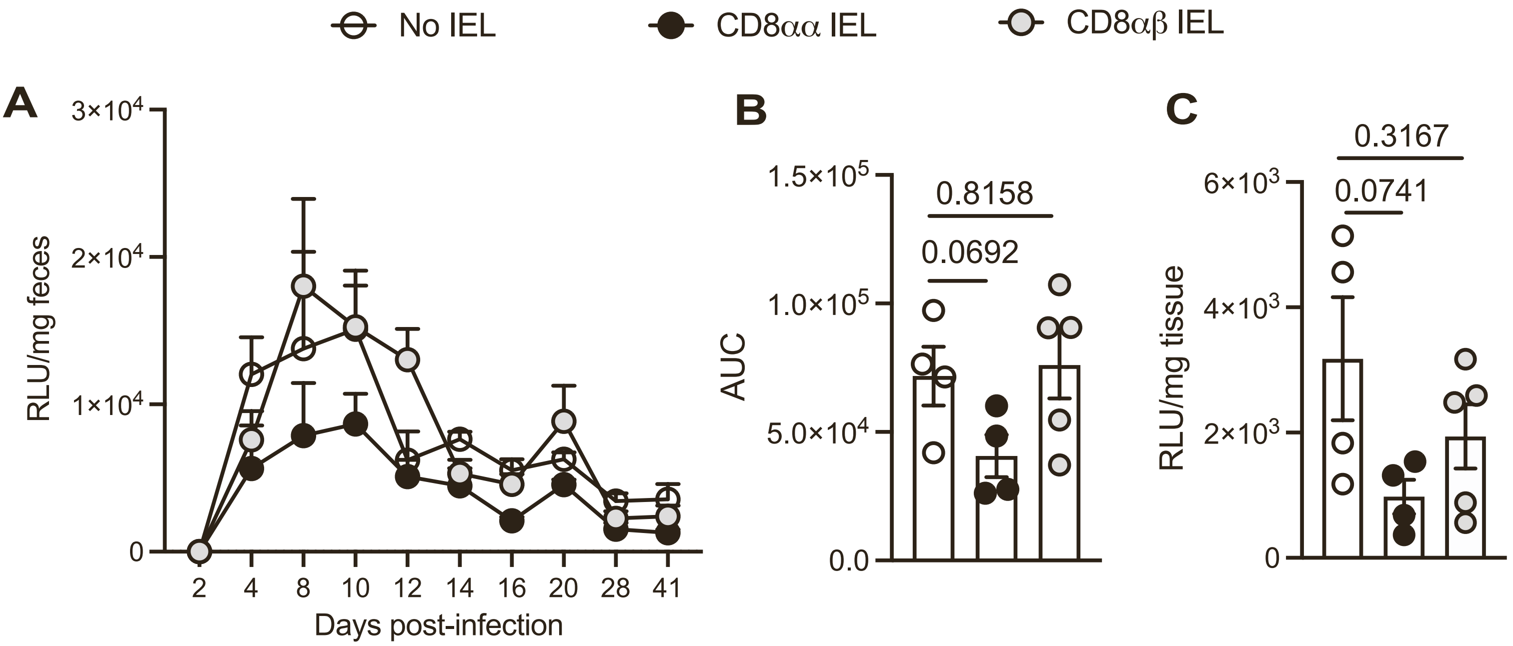

Supplementary Figure S4: C. tyzzeri parasite burden in Rag2-IL2Rg-CD47 triple knockout mice that received either CD8αα+ IELs or CD8αβ+ IELs (Related to Figure 5). (A) Ct-CR parasite burden in the fecal samples of CD8αα+ or CD8αβ+ IEL-transferred Rag2-IL2Rγ-CD47 triple knockout mice compared to mice that received no IELs over a long-term infection, and the area under the curve (B) corresponding to the same experiment. (C) Parasite burdens in the ileum of mice culled at day 41 post-infection. Each dot in (B) and (C) represents individual mice. Error bars, mean + SEM. p-values are provided, as calculated by t-test.
