# Supplementary material for: Dietary environmental factors shape the immune defense against Cryptosporidium infection
Source: Cell Host Microbe. Author manuscript; Available in PMC 2026 Mar 20. (PMC7618106; doi:10.1016/j.chom.2023.11.008)
Supplement: Table S1 [file EMS208360-supplement-Table_S1.pdf]

Supplementary Table 1: Summary of genome comparisons between *C. tyzzeri* strain UGA55 and *C. tyzzeri* strain CR2206

	SNPs		INDELs
**Coding regions**	Synonymous	Non-synonymous	2464
	3773	5014	
**Non-coding regions**	4315		518
